# Adrenal insufficiency in prolonged critical illness

**DOI:** 10.1186/cc6895

**Published:** 2008-05-08

**Authors:** Jenn-Yu Wu, Szu-Chun Hsu, Shih-Chi Ku, Chao-Chi Ho, Chong-Jen Yu, Pan-Chyr Yang

**Affiliations:** 1 Department of Internal Medicine, National Taiwan University Hospital, Chung-Shan South Road, Taipei, Taiwan 100, Republic of China; 2 Department of Laboratory Medicine, National Taiwan University Hospital, Chung-Shan South Road, Taipei, Taiwan 100, Republic of China; 3 Department of Emergency Medicine, National Taiwan University Hospital, Chung-Shan South Road, Taipei, Taiwan 100, Republic of China

## Abstract

**Introduction:**

Adrenal insufficiency is common in critically ill patients and affects their prognosis, but little is known about how adrenal function changes during prolonged critical illness. This study was conducted to investigate dynamic changes in cortisol levels in patients with critical illness who do not improve after treatment.

**Methods:**

This observational cohort study was performed in the intensive care units of a university hospital. We studied acutely ill patients with initial cortisol level above 34 μg/dl, but who did not improve after treatment and in whom follow-up cortisol levels were determined during critical illness. All clinical information and outcomes were recorded.

**Results:**

Fifty-seven patients were included. Ten patients had follow-up cortisol levels above 34 μg/dl, 32 patients had levels between 34 and 15 μg/dl, and 15 patients had levels under 15 μg/dl. Outcomes did not differ significantly among the three groups with different follow-up cortisol levels. In Cox regression analysis, those patients who survived to hospital discharge with second cortisol levels under 15 μg/dl had a longer hospital length of stay (odds ratio = 14.8, 95% confidence interval = 2.4 to 90.0; *P *= 0.004).

**Conclusion:**

The majority of acutely ill patients who remained in a critical condition had decreased serum cortisol levels. Depressed cortisol levels at follow up may lead to worse clinical outcomes. We propose that repeated adrenal function testing be conducted in patients with prolonged critical illness.

## Introduction

Elevation in corticosteroid level to meet physiological needs during acute illness is a protective response to stress. This homeostasis is maintained by the hypothalamic-pituitary-adrenal (HPA) axis [1-6]. However, inadequate response as a result of corticosteroid insufficiency is common in critically ill patients, especially those with severe sepsis or septic shock [7,8]. Thus, corticosteroids could be beneficial in the setting of septic shock or severe acute illness [8-13].

The definition of corticosteroid insufficiency varies [3,7,8,14-21]. The proposed values for diagnosis of corticosteroid insufficiency includes random cortisol levels below 15 μg/dl [3,14-18], random cortisol levels under 10 μg/dl [19,20], random cortisol level below 25 μg/dl [21], or corticotropin stimulation test with an increase under 9 μg/dl [3,7,8,18,20]. Although the corticotropin test is preferred in clinical practice, it would be more useful to identify the level below which adrenal insufficiency is more likely and above which it is unlikely. Previous studies have suggested that serum cortisol level below 15 μg/dl should be regarded as adrenal insufficiency, warranting corticosteroid supplementation [3,14-16]. On the other hand, adrenal insufficiency is not likely if the serum cortisol level is above 34 μg/dl [3]. When the diagnosis of adrenal insufficiency is established in acute illness, extended treatment with low-dose hydrocortisone may be required [8].

Once regarded as normal adrenal function, adrenal insufficiency may develop later with prolonged critical illness [22,23]. It is easily overlooked and may occur as a result of chronic secretion of systemic cytokines or of other substances that suppress the HPA axis [24]. There is still no consensus on how often adrenal function testing should be repeated, although a re-evaluation should be considered if clinical symptoms and signs suggest adrenal insufficiency or deteriorating clinical condition [22,23].

In this retrospective cohort study, we enrolled acutely ill patients admitted to the intensive care units (ICUs) whose initial serum cortisol was above 34 μg/dl and who exhibited no improvement within 2 weeks. The aim was to evaluate changes in cortisol levels in these patients and to correlate these changes with prognosis.

## Materials and methods

### Patients

All adult patients (age > 18 years) admitted to the ICUs of the National Taiwan University Hospital between January 2005 and December 2006 were surveyed. The hospital is a tertiary academic institution with a total of 141 beds in 14 ICUs. Each of the 14 ICUs had a full-time ICU intensive care specialist and at least three rotating residents. This study was approved by the hospital's institutional review board.

Patients in whom cortisol levels were determined twice in the ICU (follow-up cortisol level within 4 to 14 days after the first cortisol determination) and in whom the initial cortisol level was above 34 μg/dl were included. Patients were excluded if there was clinical improvement (defined as a decrease in Sequential Organ Failure Assessment [SOFA] between the two cortisol level tests) and if they underwent steroid treatment before the second cortisol level determination.

### Data collection

The demographic data for the patients and the etiologies of ICU admission were recorded. All of the clinical data between two serum cortisol determinations were collected, including body temperature, shock status, SOFA scores, Acute Physiology and Chronic Health Evaluation (APACHE) II scores, dosages of norepinephrine and dopamine, serum albumin levels, and cortisol levels.

The ICU physician decided whether a patient should undergo a serum cortisol test based on clinical features suggesting corticosteroid insufficiency, such as instable hemodynamic status, fever with no obvious source, hypoglycemia, and so on. Blood was drawn at 06:00 hours and cortisol assay was performed on the same day. Serum cortisol levels were measured using a commercially available solid-phase chemiluminescent immunoassay (IMMULITE^® ^2000 Cortisol; Diagnostic Products, Los Angeles, CA, USA). Hydrocortisone 50 mg intravenous every 6 hours was administered as a steroid supplement, if clinically indicated. The usage duration and rate of tapering depended on the ICU physician's judgment, based on the clinical status of the patient.

### Outcome evaluation

The primary outcome was mortality during the hospital admission. Secondary outcomes included duration of mechanical ventilation and lengths of ICU and hospital stays. According to the criteria proposed by Cooper and Steward [3], the patients were stratified into three groups, based on the second (follow-up) cortisol level: > 34 μg/dl, 34 to 15 μg/dl, and < 15 μg/dl. Outcomes were compared among these three groups. Corticotropin tests were not performed at the National Taiwan University Hospital because of the lack of available intravenously injected corticotropin, and – under such circumstances – the adrenal response to corticotropin was not used to stratify patients.

### Statistical analysis

All continuous variables were presented as medians (interquartile range [IQR]), unless otherwise specified, and were compared using nonparametric Mann-Whitney U-tests. All categorical variables were analyzed using the χ^2 ^test, except where small sample size mandated use of the Fisher's exact test. The Kaplan-Meier method and log-rank test were used to analyze time-related events between groups. We also conducted multivariate Cox regression models to adjust for confounding factors. All of the statistical tests were two-tailed, and differences at a *P *value < 0.05 were considered statistically significant.

## Results

**Figure 1:**
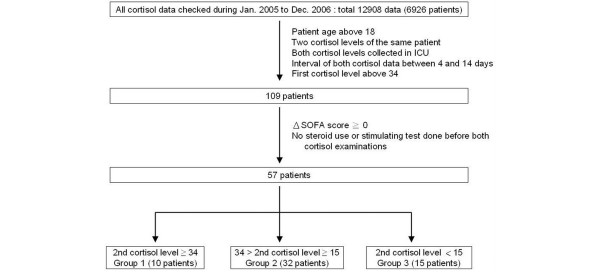
Flow chart of patient recruitment. ICU, intensive care unit; SOFA, Sequential Organ Failure Assessment.

In this cohort study, 12,908 sets of cortisol data from 6,926 patients were analyzed (Figure 1). From 109 patients who satisfied the inclusion criteria, 52 exhibited clinical improvement (decrease in SOFA score) and were excluded. Fifty-seven patients remained for analysis. Their primary reasons for ICU admission were pneumonia (40.4%), sepsis (26.3%), acute myocardial infarction (8.8%), chronic obstructive pulmonary disease (5.3%), congestive heart failure (5.3%), arrhythmia (5.3%), hypovolemic shock (5.3%), and cerebrovascular accident (3.5%). The median age was 79 years, and 33 of the 57 patients were male.

The median initial cortisol level of the study population was 42.9 μg/dl, whereas the median second (follow-up) cortisol level was 22.3 μg/dl (Table 1). The median (IQR) length of the ICU stay was 28 days (27 days), and the median (IQR) hospital stay was 41 days (48 days). Thirty-two (56.1%) of the 57 patients died. Among the remaining 25 survivors who eventually discharged from hospital, seven were ventilator dependent.

In the second cortisol level, 10 patients had cortisol levels above 34 μg/dl (group 1), 32 patients had levels between 34 and 15 μg/dl (group 2), and 15 patients had levels below 15 μg/dl (group 3). The percentage of patients with a second cortisol level below 34 μg/dl was high (47/57 [82.5%]), and adrenal insufficiency (cortisol level < 15 μg/dl) was identified in 15 out of 57 patients (26.3%; Table 1). No significant differences in etiology and other demographic factors were identified among the three groups, possibly because of the small number of patients. There were also no significant differences between the three groups in mortality, interval between the two cortisol tests, or disease severity (SOFA and APACHE II scores). Their serum albumin levels were also similar. Durations of mechanical ventilation, as well as length of ICU and hospital stay increased with lower second cortisol level (group 3 > group 2 > group 1), although the increase did not reach statistical significance. Ventilator dependence was more frequent in adrenal insufficiency (group 3), but the difference was not statistically significant.

**Table 1:** Patient characteristics

Variables	All (*n *= 57)	Group 1 (*n *= 10)	Group 2 (*n *= 32)	Group 3 (*n *= 15)	*P*
Age (years)	79 (16)	75 (20)	80 (12)	71 (25)	NS
Sex (*n*; male/female)	33/24	7/3	16/16	10/5	NS
First cortisol level (μg/dl)	42.9 (14.8)	49.3 (27.7)	41.4 (17.3)	42.0 (20.9)	NS
Second cortisol level (μg/dl)	22.3 (15.8)	45.1 (25.5)	23.4 (9.3)	9.6 (8.8)	< 0.05
Change in cortisol level (μg/dl)	24.7 (28.7)	0.8 (13.3)	21.1 (16.9)	34.8 (16.7)	< 0.05
Time interval between both cortisol tests (days)	8 (5)	8 (3)	8 (6)	9 (5)	NS
Etiologies (*n*)					
Pneumonia	23	5	11	7	NS
Chronic obstructive pulmonary disease with acute exacerbation	3	0	1	2	NS
Sepsis	15	2	8	5	NS
Hypovolemic shock	3	0	3	0	NS
Cerebrovascular accident	2	0	1	1	NS
Congestive heart failure	3	0	3	0	NS
Arrhythmia	3	1	2	0	NS
Acute myocardial infarction	5	2	3	0	NS
Diabetes mellitus (*n*)	17	4	12	1	NS
Malignancy (*n*)	12	1	10	1	NS
First albumin level (g/dL)	3.0 (0.7)	3.0 (0.6)	2.9 (1.0)	3.0 (0.7)	NS
Second albumin level (g/dL)	2.8 (0.8)	2.6 (0.5)	2.9 (1.1)	2.8 (0.6)	NS
First SOFA score	9 (5)	10 (8)	9 (6)	8 (3)	NS
Second SOFA score	10 (5)	13 (9)	10 (6)	9 (3)	NS
First APACHE II score	21 (8)	22 (11)	21 (8)	19 (9)	NS
Second APACHE II score	21 (9)	22 (10)	21 (7)	18 (10)	NS
Change in body temperature (°C)	0.0 (1.1)	0.2 (3.1)	0.0 (0.8)	0.0 (1.0)	NS
First dopamine dosage (μg/kg/min)	6.0 (11.0)	6.0 (20.0)	5.0 (4.8)	6.0 (13.0)	NS
Second dopamine dosage (μg/kg/min)	4.0 (8.0)	5.0 (10.0)	3.5 (6.8)	4.0 (9.0)	NS
First norepinephrine dosage (μg/min)	2.0 (1.0)	4.0 (4.0)	2.0 (1.0)	2.0 (1.0)	NS
Second norepinephrine dosage (μg/min)	3.0 (4.5)	3.5 (8.5)	2.0 (1.0)	4.0 (6.0)	NS
Mortality (*n *[%])	32 (56%)	6 (60%)	17 (53%)	9 (60%)	NS
Ventilator dependent (*n *[%])	7 (12%)	1 (10%)	3 (9%)	3 (20%)	NS
Mean ICU LOS (days)	28 (27)	23 (23)	26 (18)	41 (29)	NS
Mean hospital LOS (day)	41 (48)	32 (22)	35 (36)	69 (26)	NS

Unless otherwise stated, variables are presented as median (interquartile range). APACHE, Acute Physiology and Chronic Health Evaluation; LOS, length of stay; NS, nonsignificant; SOFA, Sequential Organ Failure Assessment.

**Figure 2:**
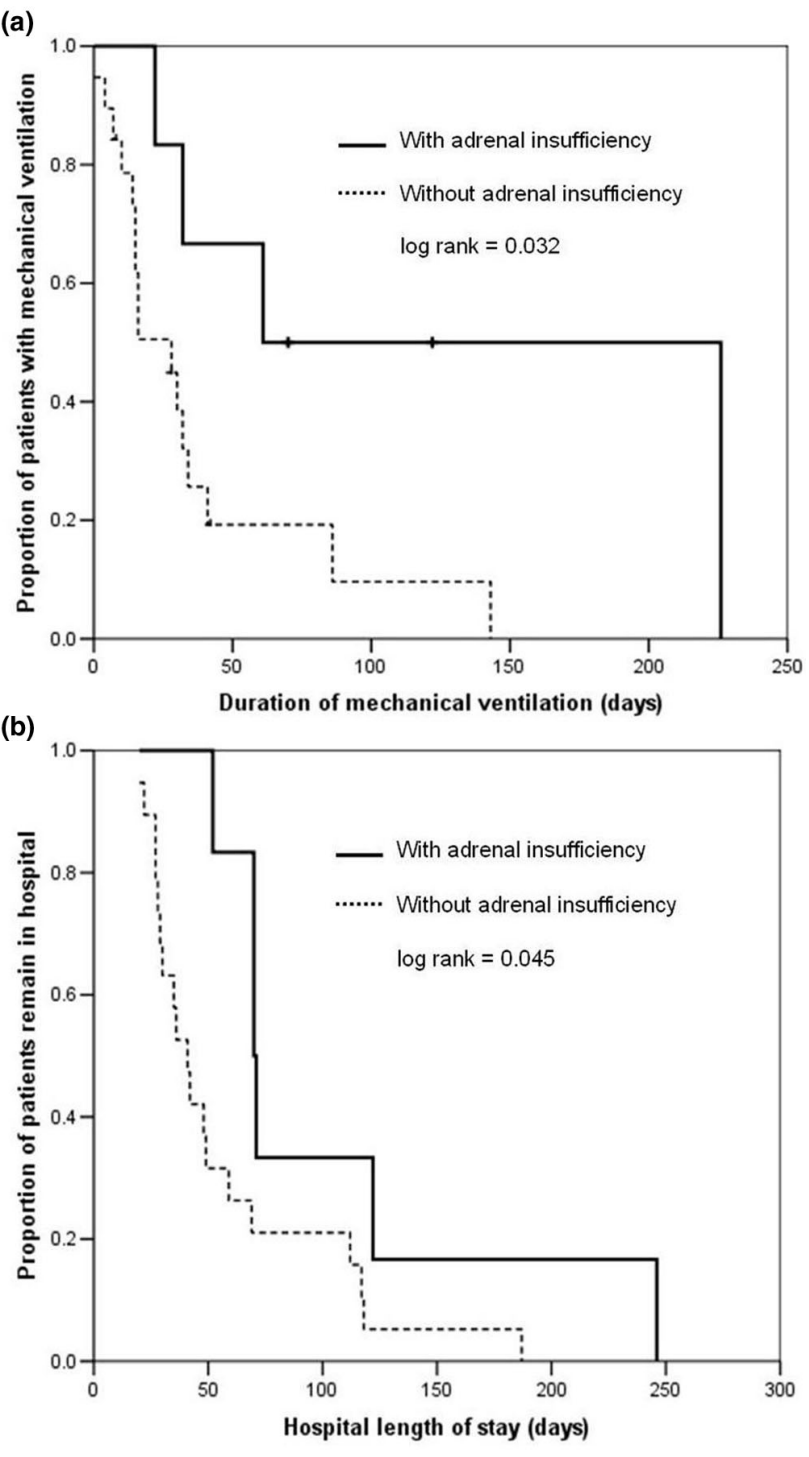
Mechanical ventilation and hospital length of stay. Shown is a comparison of **(a) **duration of mechanical ventilation and **(b) **hospital length of stay. Patients who survived to hospital discharge with adrenal insufficiency (group 3 [second cortisol level < 15 μg/dl]) had longer ventilator duration and hospital stays than did others without adrenal insufficiency (groups 1 and 2 [second cortisol level ≥15 μg/dl]), as estimated by univariate Kaplan-Meier analysis.

In subgroup analysis (Table 2), patients who survived to hospital discharge with adrenal insufficiency (group 3) had fewer ventilator-free days and longer hospital stay than other patients (groups 1 and 2), as estimated by univariate Kaplan-Meier analysis (Figures 2a, b; log-rank *P *= 0.032 and 0.045, respectively). We further conducted Cox regression models to adjust for the following confounding factors: sex, age, reason for ICU admission, and APACHE II score at ICU admission. A second cortisol level below 15 μg/dl (group 3) was not associated with a shorter ventilator-free duration in multivariate models, but it was still related to shorter hospital stay (odds ratio = 14.8, 95% confidence interval = 2.4 to 90.0; *P *= 0.004).

Eleven of 15 patients with definite adrenal insufficiency (group 3) underwent steroid supplementation (Table 3). The other four patients (three survived and one died) did not use steroids because of *Mycobacteria tuberculosis *infection and extreme hypertension. Only two patients in group 2 received steroid, based on the decision of attending physicians. We were unable to identify any effect of steroid supplementation on survival, possibly because of the small number of patients included.

**Table 2:** Outcome comparison of patients that survived to hospital discharge

	Cortisol < 15 μg/dl (*n *= 6)	Cortisol ≥15 μg/dl (*n *= 19)	Kaplan-Meier (*P*)	Cox regression^a ^(*P*)
Duration of mechanical ventilation (days)	66 (119)	16 (24)	0.032	0.055
ICU LOS (days)	37 (27)	20 (26)	0.157	0.160
Hospital LOS (days)	71 (88)	41 (41)	0.045	0.004

Values are presented as median (interquartile range). ^a^Adjusted for age, sex, etiology of ICU admission, and APACHE II score at ICU admission. ICU, intensive care unit; LOS, length of stay.

**Table 3:** Clinical features of group 3 (second serum cortisol level < 15 μg/dl)

Patient	Age/sex	First cortisol (μg/dl)	Second cortisol (μg/dl)	Outcome	Ventilator dependent	Steroid use after second cortisol	Reason why steroid treatment witheld
1	83/male	40.9	14.4	Survived	No	No	Active tuberculosis
2	58/male	95.6	14.4	Survived	No	Yes	
3	76/female	58.5	14.4	Survived	No	No	Extreme hypertension
4	79/male	34.0	12.7	Died	No	Yes	
5	70/male	40.1	11.2	Died	No	No	Active tuberculosis
6	88/female	38.6	10.6	Survived	Yes	No	Active tuberculosis
7	87/male	38.1	10.2	Died	No	Yes	
8	66/male	42.9	9.6	Died	No	Yes	
9	71/male	67.0	9.5	Survived	Yes	Yes	
10	82/female	42.0	5.6	Died	No	Yes	
11	61/male	39.2	5.3	Died	No	Yes	
12	53/female	45.3	3.9	Survived	No	Yes	
13	83/male	37.4	3.6	Died	No	Yes	
14	50/female	62.5	3.4	Died	No	Yes	
15	45/male	37.6	2.8	Survived	No	Yes	

## Discussion

The definition of adrenal insufficiency in patients with critical illness remains controversial [3,20,21]. We used the definition formulated by Copper and coworkers [3]: adrenal insufficiency is likely if a random cortisol level is under 15 μg/dl and unlikely if the cortisol level is above 34 μg/dl. We also defined adrenal insufficiency based on work reported by Lipiner-Friedman and colleagues [18], who found that patients with a cortisol level below 15 μg/dl were more likely to die, to have a longer duration of shock, and to have a shorter survival time.

Although the physiological dose of corticosteroid used for septic shock remains controversial, adrenal insufficiency continues to be an issue in critical care medicine [25-31]. Only a few reports [22,23] have focused on the time course of cortisol levels in patients with prolonged illness. In the present study we retrospectively analyzed cortisol levels in ICU patients who did not improve after treatment, and we showed that the majority of ICU patients who did not improve clinically had decreasing serum cortisol levels during follow up.

All of the patients included in the study were still critically ill at the time of the second blood draw for cortisol levels, all had relatively high APACHE II score (median = 21 [IQR = 9]) and SOFA score (median = 10 [IQR = 5]), and were frequently hypotensive because of inotrope use. Based on the suggestion of Cooper and Steward [3], 32 of the 57 (56.1%) patients underwent further evaluation for adrenal insufficiency (cortisol levels between 34 and 15 μg/dl) and 15 (26.3%) had adrenal insufficiency (cortisol levels < 15 μg/dl). Corticosteroid supplementation was warranted in these 15 patients.

Decreased cortisol levels during follow up is not unusual in critically ill patients [22,23]. Guzman and coworkers [22] identified a group of patients who had septic shock with prolonged use of inotropic agents. Repeated cortisol testing, with a mean of 6 days after initial testing, revealed decreasing serum cortisol levels in most patients. In another study, Marik and colleagues [23] found that 16% of ICU patients with liver failure developed adrenal insufficiency after initially normal cortisol levels, which they described as adrenal exhaustion. In our study, the patients received repeated cortisol testing at 4-day to 14-day (median = 8 days [IQR = 5 days]) intervals. In ICU patients who did not improve after this time interval, the physicians usually had to search for factors other than the original diagnosis. Most of the patients with prolonged critical illness included in our study exhibited a decrease in serum cortisol levels over time. Some (26.3%) even became adrenally insufficient, based on the criteria used. Adrenal function might be a dynamic process in patients with unstable hemodynamic status after initial management. Whether the decline in cortisol levels in patients with prolonged critical illness was an adaptation or an exhaustion of the HPA axis remains to be investigated [32].

In the study comparing patients with respiratory failure and different levels of adrenal function conducted by Huang and coworkers [33], patients with adequate adrenal reserve underwent successful weaning at a higher percentage than did those with adrenal insufficiency. A similar finding was identified in our study. Our study was not sufficiently powered to identify significant differences in overall mortality, duration of mechanical ventilator use, and length of ICU and hospital stay among the three groups, although we found a trend toward a longer duration of mechanical ventilator use and ICU and hospital stay in patients with lower second cortisol levels. Further analysis revealed that, in terms of survival, patients with adrenal insufficiency (group 3) had significantly longer ventilator use and hospital stay. Because adaptation and maintenance of homeostasis in the HPA axis is important in critical illness, the decreased cortisol levels observed in patients with prolonged critical illness included in our study might be due to the exhaustion of adrenal function. However, further studies with large patient numbers are necessary to address this issue rigorously in prolonged critical illness.

Corticosteroid treatment in septic shock remains controversial. Although treatments with low-dose hydrocortisone and fludrocortisone were suggested by Annane and coworkers [8], a recent study identified no benefit from 'physiologic doses' of corticosteroids in septic shock [34]. However, the durations of corticosteroid use in these two studies were only 7 days [8] and 11 days [34]. If the patients exhibited no improvement after prolonged treatment in the ICUs, then starting treatment with low-dose corticosteroid in these patients might be another issue for further study.

Our study has several limitations. First, a corticotropin test was not performed in our hospital. In this circumstance, group 2 patients (whose second serum cortisol level was between 34 μg/dl and 15 μg/dl) did not receive a corticotropin test. Second, the study population was small. We speculate that if larger numbers could be recruited, then differences in outcomes among the three groups, such as survival rate, might be significant. Third, the serum cortisol level measured in this study was total cortisol, rather than the free form of cortisol. However, there is no well documented evidence that free cortisol has superior diagnostic efficacy to total cortisol level [34]. Fourth, the decision regarding whether to test the serum cortisol level depended on the ICU physicians, and so there may be a selection bias in the study population. This was a pilot study in which we aimed to analyze adrenal function in patients with prolonged critical illness. Large trials are necessary to relate the outcomes of prolonged critical illness to relative adrenal insufficiency. High-quality randomized control trials are necessary to evaluate the efficacy of corticosteroids at 'physiologic doses' in these patients.

## Conclusion

Many ICU patients have decreased cortisol levels after prolonged treatment without clinical improvement. Whether there is an adaptation or exhaustion of adrenal function is still to be established. We propose that serum cortisol level testing be repeated in patients with prolonged critical illness, even in those whose initial adrenal function is within normal limits.

## Key messages

• Change in adrenal function is a dynamic process in ICU patients, but no guidelines are available to suggest how frequently serum cortisol level testing should be repeated.

• The present study suggests that the majority of long-stay ICU patients who did not improve clinically had decreased cortisol levels.

• The decrease in cortisol levels may lead to poorer prognosis, and more studies are required to clarify their importance.

• More data are required to draw a conclusion regarding whether steroid supplements are beneficial in ICU patients with prolonged critical illness.

## Abbreviations

APACHE = Acute Physiology and Chronic Health Evaluation; HPA = hypothalamic-pituitary-adrenal; ICU = intensive care unit; IQR = interquartile range; SOF = Sequential Organ Failure Assessment.

## Competing interests

The authors declare that they have no competing interests.

## Authors' contributions

JYW drafted the manuscript and perform the statistical analysis. SCH helped to collect the cortisol data. SCK participated in the analysis and interpretation of data. CCH conceived of the study, and helped to draft the manuscript. CJY and PCY revised the manuscript. All authors read and approved the final version of the manuscript.
